# Fætal Condition of the Lung after Birth, or Atelectasis

**Published:** 1848-04

**Authors:** 


### ECLECTIC DEPARTMENT,
### AND SPIRIT OF THE MEDICAL PERIODICAL PRESS.

---

*Fœtal Condition of the Lung after birth, or Atelectasis.*—Some fragmentary notices of a form of congenital imperfection of the lungs, to which the attention of Pathologists has been of late directed, and which has received the name of fœtal condition, or atelectasis, have been already published in this Journal. As the subject, however, is one of pathological interest and importance, and is, as yet, for the most part, unnoticed in systematic treatises, we have thought that a fuller account of it would be acceptable to our readers. The following section of the work entitled *Hasse's Pathological Anatomy*, (noticed in a previous number), presents the best description of the morbid state referred to, which has fallen under our notice. The author has bestowed upon it special attention, and is, in part, entitled to the merit of having introduced the subject to the attention of the Profession. The practitioner, if we mistake not, will find in this account, a key to the pathology of certain cases of infantile disease, in which the functions of the lungs are chiefly compromised, but without the presence of symptoms indicating a degree of inflammation commensurate with the severity and fatal progress of the disease.—[Ed. Buff. Med. Jour.

"This disease consists in the imperfect expansion of the lungs by the first inspirations after birth; that is,—in a permanence of the fœtal state, in the lung of the new-born infant. It is to be regarded as a disease dependent upon restricted functional developement at the time of birth, and not upon any original defect of formation in the respiratory organs. When at all extensive, atelectasis terminates sooner or later in death; under more favorable circumstances, however, in early and complete, or in tardy and imperfect recovery. For, during the first days after birth, it is still possible that the evil may be surmounted by the vigorous penetration of air; whereas, at a later period, organic changes supervene, which forever incapacitate the undeveloped portion of lung from performing its proper office. There is, however, still wanting a complete account of atelectasis, as regards both materials and pathological deductions. I shall now endeavor to furnish as faithful an anatomical description as the case will admit of, the result partly E. Jorg's and partly of my own research.

"An entire lung, or even an entire lobe, is seldom found in a state of atelectasis,—but for the most part only single and scattered lobules. Experience shows that certain portions of the lungs are especially prone to retain the fœtal condition, namely, the inferior lobes of both lungs, and the posterior half of the remaining lobes generally. Still examples occur of several lobules near the anterior surface being found in like manner undeveloped.

" The diseased patches display a brown-red or rather a blueish-red color, which is more intense if the whole lobule is uniformly unexpanded,—in which case, it is marked off by a sharp contour from the surrounding pale-red healthy substance. Where, on the other hand, scattered cells within such a lobule have become inflated, the violet color is interrupted here and there, and passes by a gradual transition, and without any distinct boundary, into the natural shade. A distinguishing feature of actelectasis is however this, that the above patches upon the surface of the lungs always exhibit a depression, the superincumbent pleura remaining perfectly smooth and polished. A lobe either entirely, or the most part, in this condition, is never found enlarged, but on the contrary, of much smaller dimensions than the others, and almost as collapsed as in the fœtus ; in general deeply imbedded within the thorax, and drawn towards the entrance of the bronchi and blood-vessels. Hence single diseased lobules do not attain the same elevation of surface as the healthy ones with which they are surrounded, but, as already stated, form depressions more or less considerable, so that the general aspect may be likened to the dimples created by emphysema, in adult lungs. Neither by incision nor pressure is any crepitation produced, unless where a few air-cells happen here and there to have become expanded. The same delicate reddish froth is never found here as in the healthy parts of the lung, but merely a small quantity of serous, slightly sanguinous fluid. The cut surface appears smooth,—uniform,—without a vestige of granular elevations. The whole of the diseased structure is not softened, but rather of a hard character, still without the tenacity of the healthy parts. When a patch so situate is cut off and placed in water, it sinks to the bottom.

" When atelectatic infants die a day or two after birth, it is generally possible to dilate artificially, the undeveloped parts. The depressed lobule is then seen to rise by degrees to the level of the rest, and to assume the color, permeability, and other characters of a sound lung, Up to this point, had other circumstances been favorable, perfect recovery might have taken place. Where, however, the little patients have survived for weeks or months, this inflation seldom succeeds, or only imperfectly. At this juncture, the unexpanded pulmonary cells are for the most part coherent : a remarkable fact, seeing how long the lungs continue unexpanded in the fœtus, without adhesion ever taking place. What ulterior transformations go on in the diseased parts, it is not yet satisfactorily determined ; it is, however, more than probable that not a few indurations and depressions, especially the small calcareous concretions, sometimes occurring without any obvious cause, at particular spots within the lungs, (generally at the back of the inferior lobes), are referrible in some measure to the above source. At all events, it may be observed generally that, in atelectasis, the boundary line between the diseased and the healthy substance becomes less and less distinct, in proportion as life is prolonged. At the earliest period of infantile existence the contrast is very decided, the diseased parts being immediately surrounded by pulmonary texture in all respects natural and healthy.

"In infants who had died of atelectasis, E. Jorg invariably found the foramen of the heart unclosed ; a fact confirmed by myself, but which, at that age, is not unusual. The brain was in a congested state. When death followed shortly after birth, the body had the appearance of being generally well developed, but was extensively ecchymosed ; the hands and toes were clenched ; and there was foam in front of the nostrils and of the closed mouth. Where, however, the disease had been of some standing, the body was wasted and the skin loose and wrinkled. Jorg remarks,that under these circumstances, both the affected and the adjacent parts are found inflamed, if not in a state suppuration. The details of Jorg's cases, however, clearly show them to have been complicated. Inflammation is neither necessarily nor even frequently the sequel of atelectasis, for often as I have witnessed this disease I have never met with a case of inflammation which could be directly traced to the diseased lobule. I have even seen a case of genuine pneumonia with hepatization of the inferior lobe, one portion of which being *atelectasic*, had not participated in the inflammation, but presented hard, knotty, depressed patches in the midst uf expanded, softened, hepatized substance.* Lobules retaining the fœtal condition, are quite passive in relation to other morbid processes, and especially to inflammation ; the examples given by Jorg appear rather to have been cases of real pneumonia.

"It is here requisite to state that *atelectasis*, with the characteristic features above described, is not always accurately distinguished from other diseases ; that condition having, in some instances, not been duly recognized, and, in many more, been confounded with quite dissimilar pulmonary affections. Indeed, the greatest discrepancy of opinion, or rather the greatest confusion prevails, so that I am here under the necessity, however hazardous it may appear, of submitting those discordant views to the ordeal of a critical examination. The French writers on children's diseases, who first turned their attention to the changes in the pulmonary texture of new-born infants, refer *atelectasis* chiefly, if not exclusively, to the

---

* The second case of Valleix (Cliniques des Maladies des Enfans nouveau-nes, 1838,) offers many points of resemblance with the above.

head of *pneumonie lobulaire*. Several German authors have, without further inquiry, adopted this view, (Vernon, Der Artz am Krankenbette der Kinder, Wien, 1838, vol. ii., p. 54, &c. ;) while others, admitting the peculiarities of atelectasis when associated with ordinary pneumonia, continue nevertheless to connect it, either directly or indirectly, with the same inflammatory process. It will not be difficult to show wherein lies the essential difference between the two. In atelectasis, the coloring of the diseased portions of lung always approaches more to a violet, their exterior appearing smooth and glistening, so as to contrast with the dull, brown-red surface of inflammation. In inflammation, again, the diseased portions are preternatural distended, whilst in atelectasis they are collapsed, and inferior even to the healthy texture in volume,—but susceptible, provided the disease has not lasted too long, of artificial inflation, and capable, through its means, of acquiring a perfectly natural apppearance. In inflammation, the pulmonary texture is softened, in atelectasis it is hard, and the cut surface is not granular, but smooth. Where no complication exists, the anatomical characters of a first or third stage of pneumonia are not discoverable either in or near the diseased patch; in short, we have nothing like pneumonia except the solid, non-crepitant mass, which has been confounded with the second stage of that disease, namely, with red hepatization. Where single pulmonary cells have been found dilated in the midst of an undeveloped lobule, the absence of softening, and of the peculiar, congested, humid character of its texture, offers a wide difference between it and the first stage of pneumonia. A portion of lung retaining its fœtal condition allows a little thin, dark, apparently natural blood to escape upon pressure: in the first degree of pneumonia a tolerable quantity of a turbid, bloody fluid, mingled with fibrin, and with a few minute air-vesicles,—in red hepatization, a tenacious dirty-brown reddish,—in gray hepatization, a large proportion of grayish-yellow purulent fluid may be expressed. Atelectasis usually affects both lungs,—pneumonia is, for the most part, confined to one. Finally, the secondary phenomena attendant upon pneumonia, as inflammation of the pleura and of the bronchial mucous membrane, softening of the bronchial glands, fibrinous concrements within the heart's cavities, &c., are wanting in atelectasis. But the peculiar characters of this fœtal condition of the lung are only thus marked during the first few weeks after birth; subsequently, when, as already stated, ulterior changes take place, it becomes extremely difficult to form an exact diagnosis from mere cadaveric inspection.

Jorg observed atelectasis in children, in whom the first act of breathing had been imperfectly accomplished, either because they were puny and feeble, or because they had been hurried into the world before placental respiration had been altogether suspended, and the necessity for pulmonary respiration become sufficiently potent to stimulate all the mucles of inspiration. He therefore concluded, that it was due to the inhaled air not sufficing to effect complete expansion of the lungs;* a view corroborated both by the symptoms and course of the affection. This partial introduction of air might be deemed at variance with the physical laws of respiration, inasmuch as the atmospheric pressure must necessarily distend the entire lung equably, not to the exclusion of a lobe, and, still less, to that of a lobule. The objection, however, falls to the ground, when it is considered that the operation of these laws is the result of previous muscular action. Moreover, there is a great analogy between the pathological relations of primal respiration and those of many other affections,—pleurisy, for instance, (see that article.) where the one-half of the chest—and especially in partial pleurisy, where certain portions—do not at all share in the movements of the remainder,—and where, again, after the absorption of circumscribed empyema, those very portions collapse and become totally inert, a partial deformity of the chest being the well-known result. We need, therefore, be at no loss to understand how defective breathing may originate in a merely partial activity of the intercostal or other respiratory muscles. It ought to be added, that such portions of the lungs as commonly require several forcible inspirations for their due expansion, are especially prone to remain in the fœtal condition. To conclude, it can hardly appear singular that atelectasis should be generally more extensive in the right lung, when we call to mind how the capacity of that half of the thorax is diminished by the great size of the liver in the fœtal state.

Atelectasis has been confounded with pneumonia by most writers on the diseases of children, as their own publications amply prove; for we find specified, under "infantile pneumonia," so much that is peculiar and enigmatical,—so many deviations from the same disease in the adult, as to render it obvious that two maladies of quite a dissimilar nature are brought under one rubric. Even the account they give of the appearances after death, are in many instances conclusive as to the justness of the above assertion; and that without reckoning the previous symptomatic relations,—namely, the frequent absence of true inflammatory phenomena, the want, or irregular accession of fever, and the very slender information derived from percussion and auscultation.† The existing difficulties have induced several writers to assume a double form of infantile pneumonia. Rilliet and Barthez‡ distinguish an acute and a chronic type: the first occurs in children, somewhat advanced, commencing with the symptoms of catarrh, and resembling in

* This explantion was given by J. C. G. Jorg (Professor of Midwifery at Leipsic,) in 1831, and published by his son, E. Jorg, M. D., the year following. See his dissertatio de pulmonum vitio organico, &c. Lips. 1832; and die Fœtuslunge im geborenen Kinde, &c. 1835.

† Compare Heyfeld r (Studien im Gebiete der Heilwissenschaft, vol. ii. p. 136,)—Kluge (Vereinszeitung, 1835, No. xxx.)

‡ Maladie des Enfans. Prem. partie, 1838.

general the pneumonia of adults; the chronic form, on the other hand, assails for the most part new-born infants, and agrees, in symptomatic and anatomical characters, very closely with atelectasis. Thus Billard* affirms congestion to be more common in infancy,—true hepatization at a more advanced period; and Heyfelder (l. c. p. 140) confirms the remark made at the hospital for sick children at Paris, namely, that in children beyond the sixth year, pneumonia bears a close affinity to that in the adult. But this division of cases does not remove the difficulty; because, as Gerhard has shown, atelectasis chiefly concerns infants not above twelve months old.† Cruise,‡ who very properly rejects the idea of atelectasis being a pneumonia or bronchio-pneumonia, met with genuine inflammation of the lungs in young infants very rarely, and found the after-death appearances to bear a near resemblance to those of the disease in adults. His assertion, however, that in quite young infants this inflammation assumes only exceptionally, if ever, the same form as in adults, is in contradiction with experience,—so far at least as the anatomical characters are concerned. Again, the views promulgated with reference to the organic changes affecting the pulmonary texture, further evidence the obscurity and confusion that prevail. Valleix in his otherwise highly valuable work (p. 195), on the one hand, declares the pneumonia of children (though confounding it with atelectasis) to be identical with that of adults; whilst on the other he is perplexed by certain incongruities, really belonging to atelectasis. His greatest difficulty is, to account for the "*hepatized*" patches being always hardened, instead of softened, and for their cut surface being smooth, instead of granular. In the new-born child he never met with those secondary results of pneumonia, so constant in adults, and whatever has been encountered of that nature by others, he is forced to ascribe to some other morbific source. He also pointedly adverts to his own experience, as well as to that of others, touching the rarity of pleuritic or bronchitic complication. Billard (l. c. p. 534) expressly seeks to show that this disease essentially differs from the pneumonia of adults. He says: "the pneumonia of the new-born obviously arises from stasis of the blood in the lungs, the stagnant blood operating as a foreign body," &c.; and again, "The cause of the inflammation is purely mechanical, and it is not to be wondered at that the pneumonia is very circumscribed, and indeed limited to the patches originally affected."§ Seifert (Die Bronchial pneumonie

* Tratie des Maladies des Enfans, nouv.-nes. 1833.

† Journ. des Connaiss. Medico-Chir. Sept. 1835.

‡ Uber die acute Bronchitis der Kinder, p. 118. 1839.

§ Cruveilhier appears to entertain similar opinions; although, where death occurs within a day or two after birth, he believes that the process of infiltration and inflammation must have commenced *in utero*. Out of twelve of his cases several, and of his figures Nos. 1 and 4 (livr. xv. pl. 2,) belong to *atelectasis*. He expresses himself thus cautiously concerning the disease: "Il meurt autant d'enfants nouveau-nes que d'adultes par les poumons."

der Neugeborenen, &c., 1837) in a like manner compares the state of the lungs in question with hypostasis, (where, however, softening takes place, and not hardening,) the inflammation being rather of a congestive, venous character. He assumes four grades of bronchio-pneumonia (p. 94); these do not, however, represent the same inflammatory disease in different degrees of advancement, but different forms of one and the same condition, the description of which cannot fail to recall atelectasis to mind. Cruse (l. c. p. 113) endeavors to convince him that neither hypostasis nor inflammation is present, and hints at the possibility of atelectasis; but, without pursuing the subject any farther, refers the pathological changes to bronchitis. Rilliet and Barthez come nearest to the truth, in describing under the pneumonia of the new-born a peculiar alteration, namely, carnification. Here the affected portion is commonly situate at the base of the lung; it presents a smooth, compact cut surface, which, on pressure, emits a thin sanguineous fluid; it is sometimes associated with hepatization, and then the lung resembles that of a fœtus that has not yet breathed. The manner in which the disease spreads within the lungs is opposed to the course of pneumonia, but consistent with that of *atelectasis*. Seifert generally found both lungs similarly affected, and Valleix mentions, from researches made by Vernois at the foundling hospital of Paris, that, out of 113 cases, there were 100 in which both lungs were simultaneously diseased.

Unequivocal cases of infantile pneumonia, whether lobar or lobular, such as I have myself examined, and as Kiwisch has published (Oesterreich. Medic. Jahrb. N. F. vol. xxi, Stuck 4, p. 534,) afford, on the other hand, the strongest negative grounds for establishing atelectasis as a distinct form. In the great majority of cases, Kiwisch found but one lung affected, the hepatized patches being invariably softened, and rendered cognizable from without, by a dull grayish coloring; conjointly with hepatization the other stages of pneumonia were present, including that of abscess; in every case there was pleuritic effusion more or less copious; bronchitis was seldom noticed,—endocarditis in one case; finally, percussion and auscultation always afforded the ordinary results, whilst the asphyctic and cyanotic phenomena were comparatively trivial.

In concluding these critical remarks, I consider myself entitled to draw the following inferences: New-born infants are prone to an organic affection of the lungs, altogether distinct from pneumonia, and dependent upon imperfect inspiration after birth, by many pathologists confounded with pneumonia, and by Rilliet and Barthez designated as carnification. The greater number of cases of pulmonary disease occurring at the earliest period of infantile life, and set down as pneumonia, may be looked upon as cases of atelectasis. The last assertion is, however, to be taken with some reserve, — inasmuch as, in vast lying-in or foundling hospitals

(Kiwisch), pneumonia is apt to become epidemic with new-born infants, and, under these circumstances, to attain a numerical preponderance over *atelectasis.*

